# Dynamic nomogram prediction model for diabetic retinopathy in patients with type 2 diabetes mellitus

**DOI:** 10.1186/s12886-023-02925-1

**Published:** 2023-04-28

**Authors:** Chunhui Zhang, Liqiong Zhou, Minjun Ma, Yanni Yang, Yuanping Zhang, Xu Zha

**Affiliations:** Department of Ophthalmology, The Second Affiliated Hospital of Kunming Medical University, Kunming, Yunnan Province China

**Keywords:** Type 2 diabetes mellitus, Diabetic retinopathy, Nomogram, Prediction

## Abstract

**Background:**

To develop a dynamic prediction model for diabetic retinopathy (DR) using systemic risk factors.

**Methods:**

This retrospective study included type 2 diabetes mellitus (T2DM) patients discharged from the Second Affiliated Hospital of Kunming Medical University between May 2020 and February 2022. The early patients (80%) were used for the training set and the late ones (20%) for the validation set.

**Results:**

Finally, 1257 patients (1049 [80%] in the training set and 208 [20%] in the validation set) were included; 360 (28.6%) of them had DR. The areas under the curves (AUCs) for the multivariate regression (MR), least absolute shrinkage and selection operator regression (LASSO), and backward elimination stepwise regression (BESR) models were 0.719, 0.727, and 0.728, respectively. The Delong test showed that the BESR model had a better predictive value than the MR (p = 0.04899) and LASSO (P = 0.04999) models. The DR nomogram risk model was established according to the BESR model, and it included disease duration, age at onset, treatment method, total cholesterol, urinary albumin to creatinine ratio (UACR), and urine sugar. The AUC, kappa coefficient, sensitivity, specificity, and compliance of the nomogram risk model in the validation set were 0.79, 0.48, 71.2%, 78.9%, and 76.4%, respectively.

**Conclusions:**

A relatively reliable DR nomogram risk model was established based on the BESR model.

**Supplementary Information:**

The online version contains supplementary material available at 10.1186/s12886-023-02925-1.

## Background

Diabetes mellitus (DM) is a chronic metabolic disease caused by multiple factors, where patients with type 2 DM (T2DM) account for the greatest number of those with DM [1]. Systemic complications of T2DM can greatly shorten life expectancy, leading to disability [2], and even endanger patients’ lives [3]. Diabetic retinopathy (DR) is one of the most common and serious complications of diabetic microangiopathy in T2DM. A previous study showed that the prevalence of DR varies across regions, reaching approximately 18.7% in southwest China [4].

Currently, the diagnosis of DR relies on a combination of the slit lamp, color fundus photography, and fundus fluorescein angiography (FFA) by physicians experienced in fundus disease or skilled technicians, which might reduce the accuracy of DR diagnosis in primary community hospitals [5]. In addition, the likelihood of a clinical cure for DR is low, and early detection and intervention are of essential importance for reducing DR-induced visual loss [6]. Therefore, developing a simple and feasible DR prediction tool is urgent.

It is well known that some factors are associated with DR, such as hyperglycemia (a 1% reduction in HbA1C level was reported to be associated with a 35% reduced risk of DR, 15-25% reduced risk of DR progression, 25% reduced risk of vision-threatening DR, and 1% reduced risk of blindness), diabetes duration (duration ≥ 20 years was reported to be associated with DR in 50-90% of patients), hypertension (a 10 mm Hg reduction in systolic blood pressure was reported to be associated with a 40-50% reduced risk in DR progression), cataract surgery, nephropathy, and pregnancy [4, 7–9]. A recent study described a machine learning model based on 17 variables; however,such models are complicated to use in the everyday clinical setting [10].

Therefore, this study aimed to develop a convenient and dynamic prediction model for DR using RGMS-II parameters in combination with conventional systemic risk factors.

## Methods

### Study design and populations

This retrospective study included T2DM patients from the electronic medical record system of the Second Affiliated Hospital of Kunming Medical University between May 2020 and February 2022. The inclusion criteria were: 1) > 18 years of age; 2) diagnosis of T2DM; 3) complete medical information. Patients with a history of vitreoretinal diseases such as vitreoretinal surgery, retinal laser photocoagulation, glaucoma, or poor-quality fundus photography images that affected DR assessment were excluded.

This study adhered to the Declaration of Helsinki and was approved by the Ethics Committee of the Second Affiliated Hospital of Kunming Medical University. Due to the retrospective nature of the study, the ethics committee waived the requirement for informed consent.

80% of the patients included in the early phase of this study were selected as the training set, while the other 20% of patients included in the later phase were selected as the validation set.

### Data collection

Detailed data were collected for medical records, including demographic characteristics (e.g., sex and age), laboratory indications [e.g., urine albumin-creatinine ratio (UACR), serum insulin, lipid accumulation product (LAP), and high-density lipoprotein cholesterol (HDL-C)], ambulatory glucose data, color fundus photography images, and medical history, based on previous literature and expert recommendations (Supplementary Table 1). LAP was calculated according to the formulas described by Kahn [11]: LAP male = [waist circumference (cm) − 65] × TG (mmol/L), LAP female = [waist circumference (cm) − 58] × TG (mmol/L). For relatively thin patients (waist circumference < 65 and 58 cm for men and women, respectively), waist circumference was manually modified to 66 cm and 59 cm, respectively, to avoid negative LAP values. The duration of DM was determined according to clinical experience. The age of onset of T2DM (AOO) was classified as described by Yeung et al. [12], and AOO ≤ 40 years was classified as early-onset DM. The diastolic blood pressure (DBP) and systolic blood pressure (SBP) were calculated as the mean value of the first 3 days of hospitalization and were classified as normal blood pressure, high normal blood pressure, and hypertension according to the 2018 Chinese Guidelines for the Management of Hypertension [13]. Urinary albumin abnormality was classified according to the American Diabetes Association (ADA) criteria [14].

DR was diagnosed according to the 2002 International Clinical Diabetic Retinopathy Disease Severity Scale [15]. Nonproliferative abnormalities included microaneurysms, intraretinal hemorrhage, venous beading, and intraretinal microvascular abnormalities. Proliferative abnormalities included neovascularization, vitreous hemorrhage, and anterior retinal hemorrhage (Supplementary Fig. 2). This study used a 45˚ 6.3-megapixel digital wheal-free camera (Canon, Japan) to acquire images of each eye, separately for the macula and the center of the optic disc. In order to ensure the accuracy of the input data, all data and fundus photography images were independently collected by two clinicians and checked and summarized by a third clinician.

### Statistical analysis

SPSS 23.0 (IBM, Armonk, NY, USA) and R 4.1.1 software (The R Project for Statistical Computing, www.r-project.org) were used for statistical analysis. Continuous data that conformed to normal distribution were described as means ± SD, and compared with the independent t-test. Continuous data with skewed distribution were described as median [interquartile range (IQR)] and compared using the Mann-Whitney U-test. Categorical data were described as frequencies (percentages) and analyzed using the chi-square test. Pearson and Spearman correlation analyses were used to explore the correlations between independent variables. Univariable logistic regression analyses were used to assess the significance of independent variables in the training set. The variables with statistical significance (P < 0.05) were analyzed by multivariable logistic regression to establish the multivariate regression (MR) model. The “glmnet” package was used for least absolute shrinkage and selection operator (LASSO) variable selection. The “glm” function and the “MASS” package were used in the backward mode to establish the backward elimination stepwise regression (BESR) model. The receiver operating characteristics (ROC) of the DR prediction model were compared using Delong’s test. The “rms” package was used to plot the DR nomogram risk model according to the DR prediction model with the highest prediction value. All statistical analyses were two-sided, and P < 0.05 was considered statistically significant.

## Results

### Characteristics of the patients

**Fig. 1:**
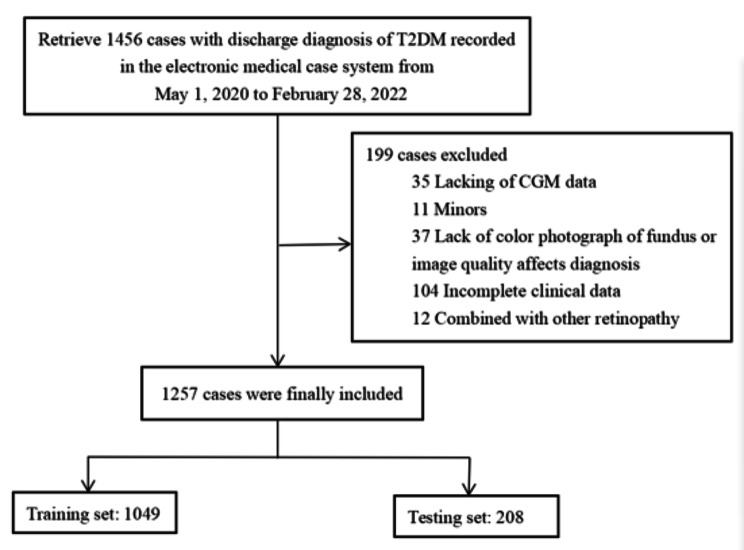
Flowchart illustrating sample selection

A total of 1456 T2DM patients were screened, of which 199 (13.7%) were excluded due to incomplete medical records, and 1257 (86.3%) patients were finally included: 468 (37.2%) women and 789 (62.8%) men (p = 0.393). DR was found in 297 (28.0%) patients in the training set (n = 1049) and 66 (31.7%) in the validation set (n = 208) (Table 1; Fig. 1).

**Table 1:** The demographic and clinical characteristics of participants

Variables	Training (n = 1049)	Validation (n = 208)	P
Age (years)	56.00 (55.59–56.99)	56.00 (53.78–57.22)	0.567
Waistline (cm)	90.00 (90.10-91.23)	90.00 (89.52–92.18)	0.946
HbA1c (%)	9.60 (9.61–9.90)	9.95 (9.53–10.12)	0.153
FPG (mmol/L)	9.48 (10.10-10.62)	9.61 (9.55–10.61)	0.930
Serum insulin (µIU/ml)	9.80 (11.25–12.40)	10.10 (10.07–12.26)	0.383
C-Peptide (ng/ml)	2.10 (2.20–2.35)	2.12 (2.14–2.50)	0.930
HOMA-IR	4.24 (5.16–5.79)	4.08 (4.60–5.89)	0.714
HOMA-beta	33.07 (34.75–80.68)	31.13 (38.98–59.46)	0.411
Fructosamine (µmol/L)	384.00 (389.73-401.93)	378.5 (374.43-401.05)	0.622
BHA (mmol/L)	0.07 (0.16–0.25)	0.07 (0.16–0.24)	0.748
TC (mmol/L)	4.82 (4.79–4.95)	4.79 (4.88–5.28)	0.949
TG (mmol/L)	1.90 (2.56–2.95)	2.10 (2.88–4.13)	0.184
HDL-C (mmol/L)	1.05 (1.08–1.16)	1.06 (1.05–1.13)	0.872
LDL-C (mmol/L)	2.99 (2.98–3.10)	2.98 (2.93–3.19)	0.970
NONHDL (mmol/L)	3.79 (3.76–3.91)	3.77 (3.80–4.18)	0.949
Lp(a) (mg/dL)	7.90 (13.59–15.76)	7.85 (12.01–16.77)	0.969
APOA1 (g/L)	1.31 (1.29–1.34)	1.21 (1.19–1.25)	0.000
APOB (g/L)	0.92 (0.92–0.97)	0.96 (0.99–1.46)	0.088
LAP (cm·mmol/L)	54.60 (74.87–88.20)	61.75 (82.44-122.16)	0.193
UREA (mmol/L)	5.26 (5.30–5.53)	5.60 (5.48–6.04)	0.003
CREA (µmol/L)	65.00 (67.04–69.90)	345.00 (349.13-360.78)	0.004
UA (µmol/L)	345.00 (349.13-360.78)	353.50 (345.65-373.35)	0.397
SD	2.24 (2.44–3.88)	2.40 (2.41–2.76)	0.088
MAGE	3.35 (3.24–7.07)	2.88 (3.01–3.76)	0.108
MG	10.15 (10.45–11.36)	10.91 (10.64–11.56)	0.004
LAGE	13.82 (14.74–20.79)	13.71 (13.76–15.97)	0.970
MODD	2.56 (2.94–5.09)	2.53 (2.69–3.11)	0.676
SBP (mmHg)			0.498
< 120	370 (35.3)	75 (36.1)	
120–139	462 (44.0)	84 (40.4)	
≥ 140	217 (20.7)	49 (23.6)	
DBP (mmHg)			0.178
< 80	625 (59.6)	110 (52.9)	
80–89	253 (24.1)	61 (29.3)	
≥ 90	171 (16.3)	37 (17.8)	
BMI (kg/m^2^)			0.732
≤ 18.49	14 (1.3)	2 (1.0)	
18.5-23.99	432 (41.2)	90 (43.2)	
24-27.99	436 (41.6)	79 (38.0)	
≥ 28	167 (15.9)	37 (17.8)	
UACR (mg/g cr)			0.005
< 30	803 (76.5)	137 (65.9)	
30–300	177 (16.9)	50 (24.0)	
> 300	69 (6.6)	21 (10.1)	
MA (mg/dl)			0.290
< 20	971 (92.6)	190 (91.4)	
20–200	70 (6.7)	14 (6.7)	
> 200	8 (0.7)	4 (1.9)	
Diabetes duration (years)			0.328
≤ 9	694 (66.2)	129 (62.0)	
10–19	255 (24.3)	56 (26.9)	
20–29	89 (8.5)	18 (8.7)	
≥ 30	11 (1.0)	5 (2.4)	
Treatment			0.927
No	264 (25.2)	50 (24.0)	
Insulin	200 (19.1)	37 (17.8)	
Oral antidiabetic drugs	354 (33.7)	72 (34.6)	
Combination therapy	231 (22.0)	49 (23.6)	
Sex			
Male	653 (62.2)	136 (65.4)	0.393
Female	396 (37.8)	72 (34.6)	
AOO (years)			0.030
≤ 40	248 (23.6)	64 (30.8)	
> 40	801 (76.4)	144 (69.2)	
Education			0.203
Below high school	449 (42.8)	99 (47.6)	
High school or above	600 (57.2)	109 (52.4)	
Family history	408 (38.9)	59 (28.4)	0.004
Smoking	395 (37.7)	67 (32.2)	0.137
Drinking	313 (29.8)	56 (26.9)	0.399
Hypertension	464 (44.2)	94 (45.2)	0.799
Hyperlipidemia	708 (67.5)	145 (69.7)	0.531
Ketosis	214 (20.4)	39 (18.8)	0.588
Kidney disease	241 (23.0)	79 (38.0)	< 0.001
CHD	241 (15.3)	33 (15.9)	0.72
Thyroid disease	623 (59.4)	140 (67.3)	0.033
Urine sugar	688 (65.6)	149 (71.6)	0.091

Categorical variables are expressed as frequencies (%), and quantitative variables are expressed as medians (IQR). (AOO: Age of onset of disease; SBP: Systolic blood pressure; DBP: Diastolic blood pressure; FPG: Fasting plasma glucose; HbA1c: Glycosylated hemoglobin; HDL-C: High-density lipoprotein; LDL-C: Low-density lipoprotein; TC: Total cholesterol; TG: Triglycerides; Lp(a): Lipoprotein (a); APOA1: Apolipoprotein A1; APOB: Apolipoprotein B; CREA: Creatinine; UA: Uric acid; UACR: Urine microalbumin/urine creatinine; MA: Microalbumin; BHA: β-Hydroxybutyric acid; HOMA-IR: Homeostatic model assessment of insulin resistance; HOMA-beta: Homeostatic model assessment of β-cell function; SD: standard deviation; MAGE: Mean amplitude of glucose excursions; LAGE: Largest blood glucose fluctuation; MODD: Mean of daily differences; CHD: coronary heart disease)

### Establishment of DR prediction model

**Fig. 2:**
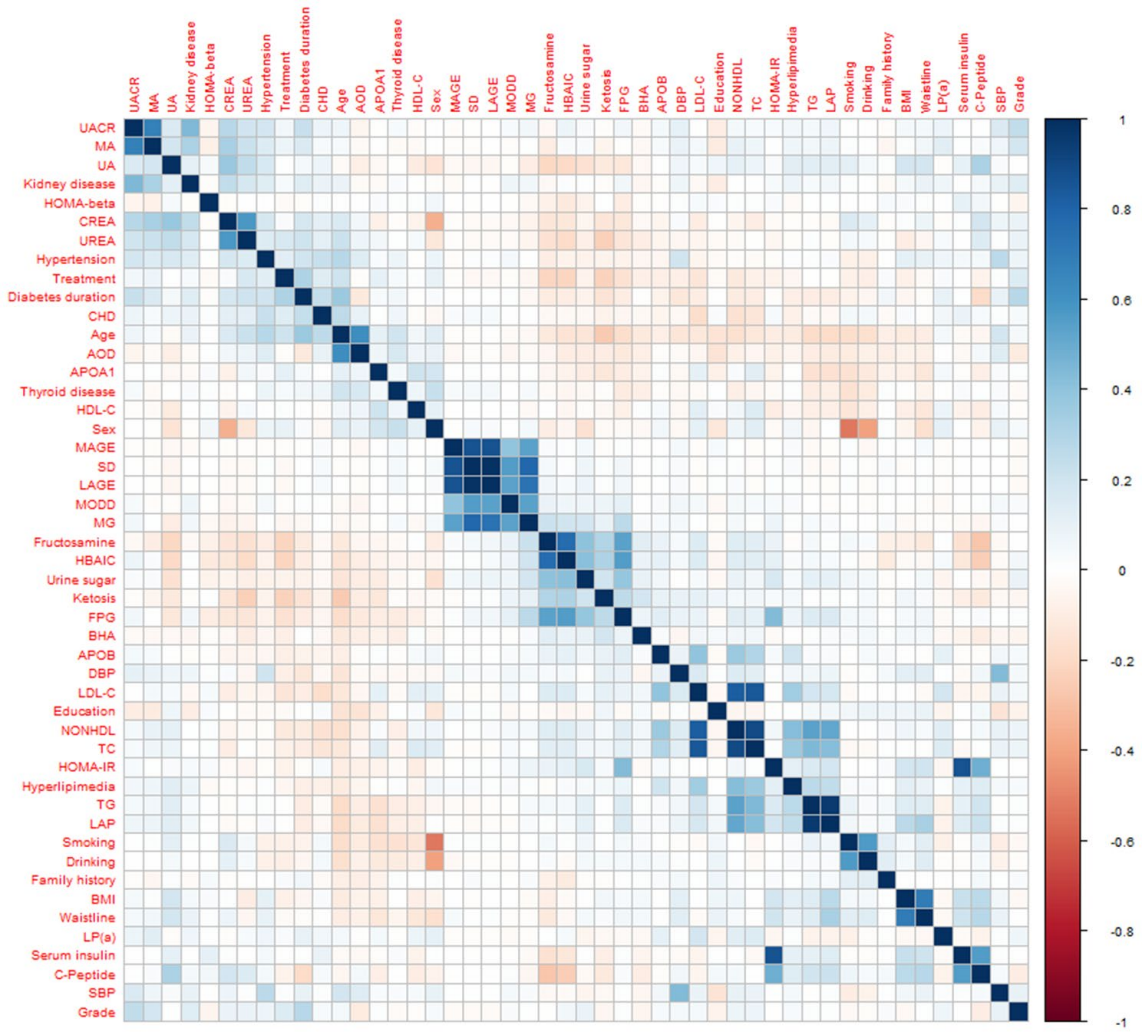
Correlation heatmap analysis of independent variables in the training set

Figure 2 and Supplementary Table 2 show the correlations between variables. Univariable regression analysis was performed first (Supplementary Table 3), and the variables associated with DR were analyzed by MR (Table 2). The results showed that longer duration of disease (10–19 years vs. <10 years: OR = 1.64 [1.14–2.34]; 20–29 years vs. <10 years, OR = 3.64 [2.13–6.26]), treatment (insulin vs. no treatment: OR = 2.48 [1.49–4.19]; oral antidiabetic drugs vs. no treatment: OR = 2.01 [1.28–3.22]; combination therapy vs. no treatment: OR = 2.38 [1.43–4.01]), UACR (UACR 30–300 vs. <30: OR = 1.82 [1.20–2.73]; UACR > 300 vs. <30: OR = 2.91 [1.07–8.14]), and urine sugar positivity compared to negative (OR = 1.65 [1.20–2.28]) were potential risk factors for the development of DR, while AOO > 40 years (OR = 0.65 [0.46–0.92]) was a potential protective factor for concomitant DR. The independent variables screened by the LASSO model were disease duration (OR = 1.99 [1.61–2.46]), treatments (OR = 1.23 [1.06–1.42]), TC (OR = 1.18 [1.06–1.32]), UACR (OR = 2.04 [1.61–2.57]), and urine sugar positive (OR = 1.65 [1.20–2.28]) (Table 2 and Supplementary Fig. 1).

**Table 2:** DR prediction models

Characteristics	Events	MR model		LASSO model		BESR model	
		OR (95%CI)	P	OR (95%CI)	P	OR (95%CI)	P
Diabetes duration (vs. <10 years)	694/139			1.99 (1.61–2.46)*	< 0.001		
10–19	255/97	1.64 (1.14–2.34)	0.007			1.83 (1.28–2.61)	< 0.001
20–29	89/52	3.64 (2.13–6.26)	< 0.001			4.11 (2.44-7.00)	< 0.001
AOO (vs. <40 years)	801/203						
> 40	248/91	0.65 (0.46–0.92)	0.016			0.68 (0.48–0.97)	0.030
Treatment (vs. No treatment)	264/34			1.23 (1.06–1.42)*	0.005		
Insulin	200/82	2.48 (1.49–4.19)	0.001			2.52 (1.52–4.25)	< 0.001
Oral antidiabetic drugs	354/93	2.01 (1.28–3.22)	0.003			1.84 (1.17–2.94)	0.009
Combination therapy	231/85	2.38 (1.43–4.01)	0.001			2.27 (1.37–3.79)	0.002
UACR (vs. <30)	803/179			2.04 (1.61–2.57)*	< 0.001		
30–300	177/73	1.82 (1.20–2.73)	0.004			1.87 (1.28–2.73)	0.001
> 300	69/42	2.91 (1.07–8.14)	0.038			3.49 (2.00-6.15)	< 0.001
Urine sugar (vs. negative)	361/83			1.65 (1.20–2.28)*	0.002		
Positive	688/211	1.65 (1.19–2.31)	0.003			1.54 (1.10–2.16)	0.013
TC (mmol/L)				1.18 (1.06–1.32)*	0.002	1.18 (1.06–1.32)*	0.003

Model 1: Multivariable Regression [MR]; Model 2: Least Absolute Shrinkage and Selection Operator Regression [LASSOR]; Model 3: Backward Elimination Stepwise Regression [BESR] *: included in the multivariable analysis as a continuous variable. OR: odds ratio; AOO: Age of onset of disease; UACR: Urine microalbumin/urine creatinine; TC: total cholesterol

The BESR was used to select the best model according to the minimal Akaike information criterion (AIC, 1111.39), which included 11 variables. After excluding non-significant independent variables, the remaining six variables independently associated with DR and included in the final model (Table 2) were longer disease duration (10–19 years vs. <10 years: OR = 1.83 [1.28–2.61]; and 20–29 years vs. <10 years: OR = 4.11 [2.44-7.00]), AOO > 40 years (OR = 0.68 [0.48–0.97]), treatment method (insulin vs. no treatment: OR = 2.52 [1.52–4.25]; oral antidiabetic drugs vs. no treatment: OR = 1.84 [1.17–2.94]; and combination therapy vs. no treatment: OR = 2.27 [1.37–3.79]), TC (OR = 1.18 [1.06–1.32]), UACR (UACR 30–300 vs. <30: OR = 1.87 [1.28–2.73]; and UACR > 300 vs. <30: OR = 3.49 [2.00-6.15]), and urine sugar positive (OR = 1.54 [1.10–2.16]).

**Fig. 3:**
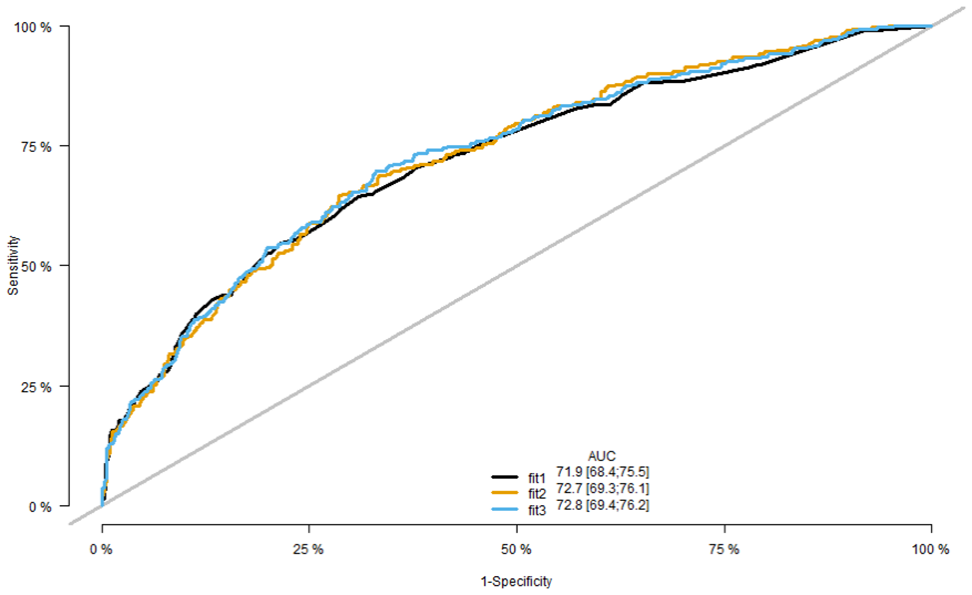
The comparison regarding area under receiver operating curves of the three regression clinical models in the training set. Fit1: Multivariable Regression Analysis; fit2: Least Absolute Shrinkage and Selection Operator Regression Analysis; fit3: Backward Elimination Stepwise Regression Analysis; ROC: receiver operator characteristic; AUC: area under the ROC curve

The ROC curves for the three regression models were plotted (Fig. 3). The AUCs for MR, LASSOR, and BESR were 0.719 (0.684, 0.755), 0.727 (0.693, 0.761), and 0.728 (0.694, 0.762), respectively. The Delong test showed that the BESR model had a better predictive value than the MR (P = 0.04899) and LASSO (P = 0.04999) models, indicating that the BESR model had the highest ability to predict DR.

### Construction and verification of the DR nomogram risk model

**Fig. 4:**
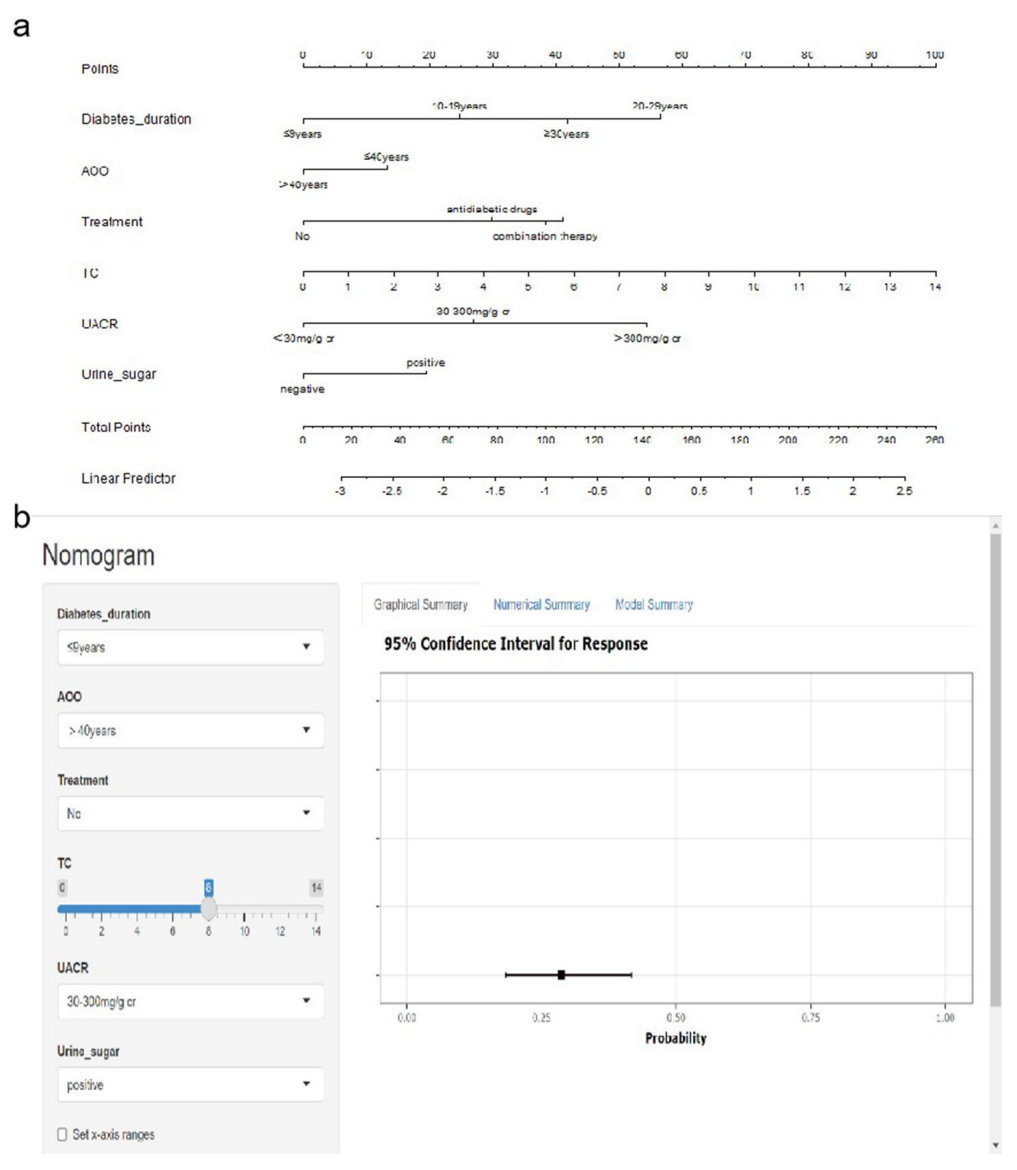
**(a)** Nomogram Prediction Model for DR in the training test. **(b)** Dynamic Nomogram Prediction Model for DR in the training set. https://yixueyanjiu.shinyapps.io/DynNomapp/

The nomogram risk model was established according to the BESR model (Fig. 4). In the validation set, the nomogram showed high predictive performance (Table 3). Specifically, the AUC, kappa coefficient, optimal cutoff, sensitivity, specificity, and compliance were 0.79, 0.48, 0.35, 71.2%, 78.9%, and 76.4%, respectively. The McNemar test showed no significant difference between the positivity rate reported by the prediction model and the actual positivity rate derived from the diagnostic criteria (P = 0.152).

**Table 3:** **The predictive power of the nomogram p**rediction model for DR in the testing set

	AUC	Kappa	Youden index	Cutoff	Sensitivity	Specificity	Diagnose accordance rate	McNemar
Testing set	0.79	0.48	0.50	0.35	71.2%	78.9%	76.4%	2.04

AUC: area under the curve

## Discussion

Over recent years, there has been an increasing number of studies on clinical prediction models for DR [10, 17–19]. However, the complexity of the pathogenesis of DR and the heterogeneity of the population limit the clinical dissemination of relevant research results. In this study, the BESR-based model showed high predictive performance in an independent validation set. The model indicated that younger AOO, long disease duration, history of insulin or oral antidiabetic drug therapy, elevated TC, elevated UACR, and positive urine sugar were independent risk factors for developing DR in patients with T2DM. This study might help improve the efficiency of DR screening and benefit more patients with T2DM. In the past, it was the view was believed that T2DM is a metabolic disease mainly associated with aging [20]; however, due to increasing social pressures and lifestyle changes, T2DM has begun to occur at an ever younger age, which has become a global trend, especially in the Chinese population with the highest disease burden of T2DM [21]. Most studies have shown that a younger age of T2DM onset is associated with an increased risk of DR [22, 23]. The results of the present study showed that late-onset DM (i.e., AOO > 40 years) [24] was a protective factor for DR. In their study, Huang et al. [23] confirmed that a younger AOO of T2DM increases the risk of DR and that such patients might also have metabolic disorders of glucose, lipids, and amino acids due to long-term hyperglycemia. The “high glucose metabolic memory” after returning blood glucose to normal levels can induce and exacerbate the inflammatory response through the histone deacetylase sirtuin-1 (SIRT1)-mediated LKB1/AMPK/ROS signaling pathway and promote apoptosis of retinal vascular endothelial cells [25]. The duration of the disease is a non-modifiable risk factor. This study showed a 1.83-fold increased risk of DR in T2DM patients with a disease duration > 10 years and a further increased risk of DR in patients with a disease duration > 20 years, thus confirming the key role of T2DM disease duration in DR progression. Bek [22] retrospectively investigated the relationship between the course of T2DM and the onset and progression of DR in 17,461 patients and showed that the risk of DR was very low in the first 10 years after onset; however, in patients with a disease course > 10 years, the risk of DR increased with time after the onset of T2DM. In order to reduce the occurrence of blinding DR, age should be moderately limited at the screening to help identify the presence of retinal microvascular damage early so that patients can be provided with appropriate intervention or guidance early in the course of DR.

As a chronic metabolic disease, conventional treatment strategies for T2DM include oral antidiabetic drugs, insulin injections, and lifestyle interventions. Still, it remains unclear whether the various treatments promote microvascular complications in patients with T2DM. Metformin is the first-line drug for treating T2DM [26], inhibiting retinal neovascularization and inflammation [27]. This study showed an increased risk of DR development in patients treated with oral antidiabetic drugs but also in patients treated with insulin. These results suggest that the treatment of T2DM is also a risk factor for developing DR. In agreement with our findings, Alemu et al. [28] showed that patients treated with insulin only had a higher risk of DR than T2DM patients treated with glycemic combination therapy. Bain et al. [29] suggested that the occurrence of DR is related to the “permeability theory”. When poor glycemic control rapidly changes to tight control, with the rapid decrease of HBA1c, intravascular osmotic pressure decreases sharply and the gradient of intra- and extracellular osmotic pressure increases, leading to changes in the retinal microvasculature, which is sensitive to pressure changes and tends to result in retinal edema. However, some scholars believe that this theory is not a good explanation for the proliferative changes in DR patients and have proposed the “synergistic hypothesis” [30]. This theory suggests that exogenous insulin acts synergistically with VEGF to cause proliferative changes in retinal microvasculature. In vitro studies also seem to support the hypothesis that Nox4-derived ROS regulate insulin-induced hypoxia-inducible factor-1α (HIF-1a) and VEGF expression through PI3K/AKT and ERK1/2 pathways; however, increased VEGF levels are involved in neovascularization and blood-retinal barrier disruption, leading to microangioma formation and vascular leakage [31]. Since all subjects in this study were hospitalized, most patients had suboptimal glycemic control. Therefore, we hypothesized that insulin therapy is associated with a high risk of developing DR and may be associated with a rapid reduction in blood glucose. Paradoxically, the early Diabetes Control and Complications Trial (DCCT) [32] looked at 726 patients with T2DM without DR and 715 patients with T2DM and mild-to-moderate nonproliferative diabetic retinopathy (NPDR) and showed a relatively higher risk of DR development and progression in the intensive treatment group; however, at subsequent longer follow-up, the risk of DR progression was lower in the intensive treatment group than in the conventional treatment. In addition, once DR progression occurred, visual improvement was more pronounced in the patients in the intensive treatment group. Therefore, it could be hypothesized that insulin treatment is associated with a high risk of DR development and possibly related to the rapid reduction of blood glucose [33]. Future studies should examine these hypotheses in order to further confirm the relationship between diabetes treatment and DR risk. DM and diabetic nephropathy (DN) are the two most common microvascular complications in patients with T2DM, and there are many similarities in the pathogenesis and progression between DN and DR [34]. The present study showed high UACR and urine sugar positivity were risk factors for developing DR in patients with T2DM. Romero et al. [35] conducted a 10-year prospective study showing that both eGFR and UACR were important risk factors for DR, with UACR having a more prominent effect on DR than eGFR. In addition, the levels of other renal function-related indicators, such as urea, creatinine, uric acid, and urine metal analysis, were higher in the DR group than in the NDR group in the univariable analyses, which could also indicate the possibility of renal injury. Therefore, a regular review should be performed for the early detection of diabetic microvascular complications.

Furthermore,our results showed that the BESR model had a significantly higher predictive value than the MR and LASSO models and that the BESR model had the highest ability to predict DR. The nomogram showed high predictive performance of DR, which is consistent with previous studies [17, 18]. The nomogram by Li et al. [17] included diabetic peripheral neuropathy (DPN), age, neutrophilic granulocyte (NE), HDL-C, hemoglobin A1c (HbA1C), duration of T2DM, and glycosylated serum protein (GSP). Still, GSP is not routinely measured in many hospitals, limiting the applicability of the nomogram. Chen et al. [18] developed a nomogram for DR based on age, diabetes duration, HbA1c, albuminuria, and triglycerides, all of which are routine variables;however, their AUC was lower than ours. Although the predictive performance of the model developed in this study was not particularly excellent (Table 4), an online DR prediction tool based on several easily accessible clinical indicators may help to improve the efficiency of DR screening and benefit more patients with T2DM (https://yixueyanjiu.shinyapps.io/DynNomapp/).

**Table 4:** Comparison with other previous validation set DR prediction models

Author	Algorithm	Number of samples	Accuracy	Sensitivity	Specificity	ROC-AUC
Li W et al(10)	XGBoost	32,452	0.900	0.700	0.900	0.900
	SVM	32,452	0.890	0.450	0.900	0.790
	LR	32,452	0.860	0.590	0.860	0.830
	RF	32,452	0.920	0.630	0.920	0.870
Cichosz et al(19)	linear classification	266	-	-	-	0.740
Li Y et al(17)	LASSO + LR	13,980	-	0.804	0.848	0.870
Chen X et al(18)	Cox regression	303	-	-	-	0.854(3 years)
		303	-	-	-	0.845(4 years)
		303	-	-	-	0.798(5 years)
The BESR of this study	LR	1257	0.764	0.712	0.789	0.790

XGBoost, extreme gradient boosting; SVM, support vector machine; LR, logistic regression; RF, random forest; ROC-AUC, areas under receiver operator characteristic curves

There are some limitations in this study. Although the data used to validate the prediction model in this study were independent of the modeling data, patient selection bias might exist since the data were from a single medical center. In future studies, we plan to collaborate with other centers and use their clinical data to conduct a more extensive and in-depth external validation of the prediction model.Moreover, although the GV parameters were calculated and analyzed, they were not included in the final nomogram risk model. The association between GV and DR needs to be further explored.

## Conclusions

A relatively reliable DR nomogram risk model was established according to the BESR model, including disease duration, age at onset, treatment method, total cholesterol, UACR, and urine sugar.

## Electronic supplementary material

Below is the link to the electronic supplementary material.

Supplementary Material 1

Supplementary Material 2

Supplementary Material 3

Supplementary Material 4

## Data Availability

All data generated or analyzed during this study are included in this published article [and its supplementary information files].

## Abbreviations

DM: Diabetes mellitus
T2DM: Type 2 DM
DR: Diabetic retinopathy
MR: Multivariate Regression
BESR: Backward elimination stepwise regression
FFA: Fundus fluorescein angiography
UACR: Urine albumin-creatinine ratio
LAP: Lipid accumulation product
HDL-C: High-density lipoprotein cholesterol
DBP: Diastolic blood pressure
SBP: Systolic blood pressure
ADA: American Diabetes Association
LASSO: Least absolute shrinkage and selection operator
ROC: Receiver operating characteristics
NPDR: Nonproliferative diabetic retinopathy
DN: Diabetic nephropathy
NE: Neutrophilic granulocyte
GSP: Glycosylated serum protein
