# Supplementary figures and images for: Dynamic nomogram prediction model for diabetic retinopathy in patients with type 2 diabetes mellitus
Source: BMC Ophthalmol. 2023 Apr 28;23:186. doi: 10.1186/s12886-023-02925-1 (PMC10142167; doi:10.1186/s12886-023-02925-1)

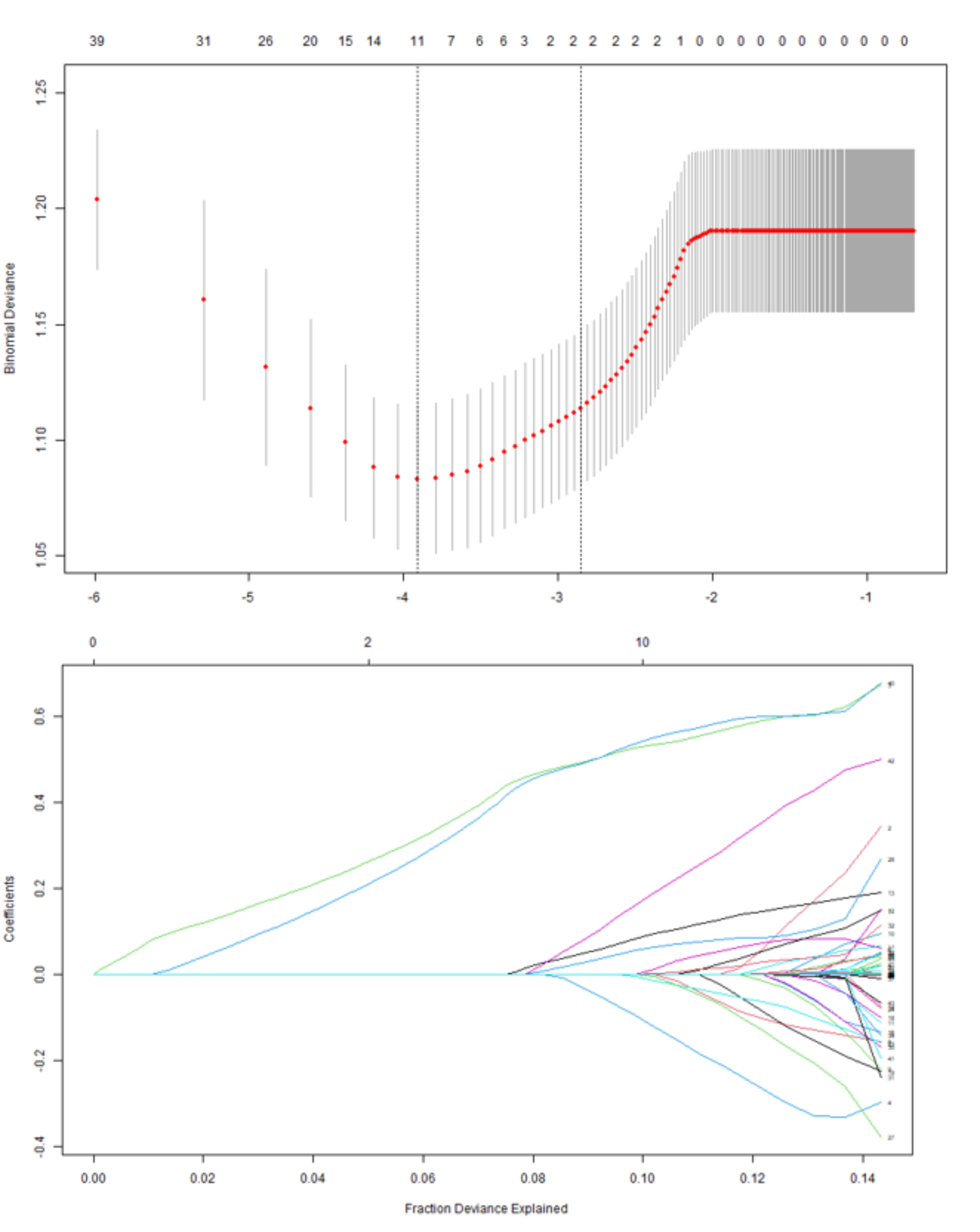

Supplement: Supplementary file 1 — Supplementary Material 1 [file 12886_2023_2925_MOESM1_ESM.jpg]

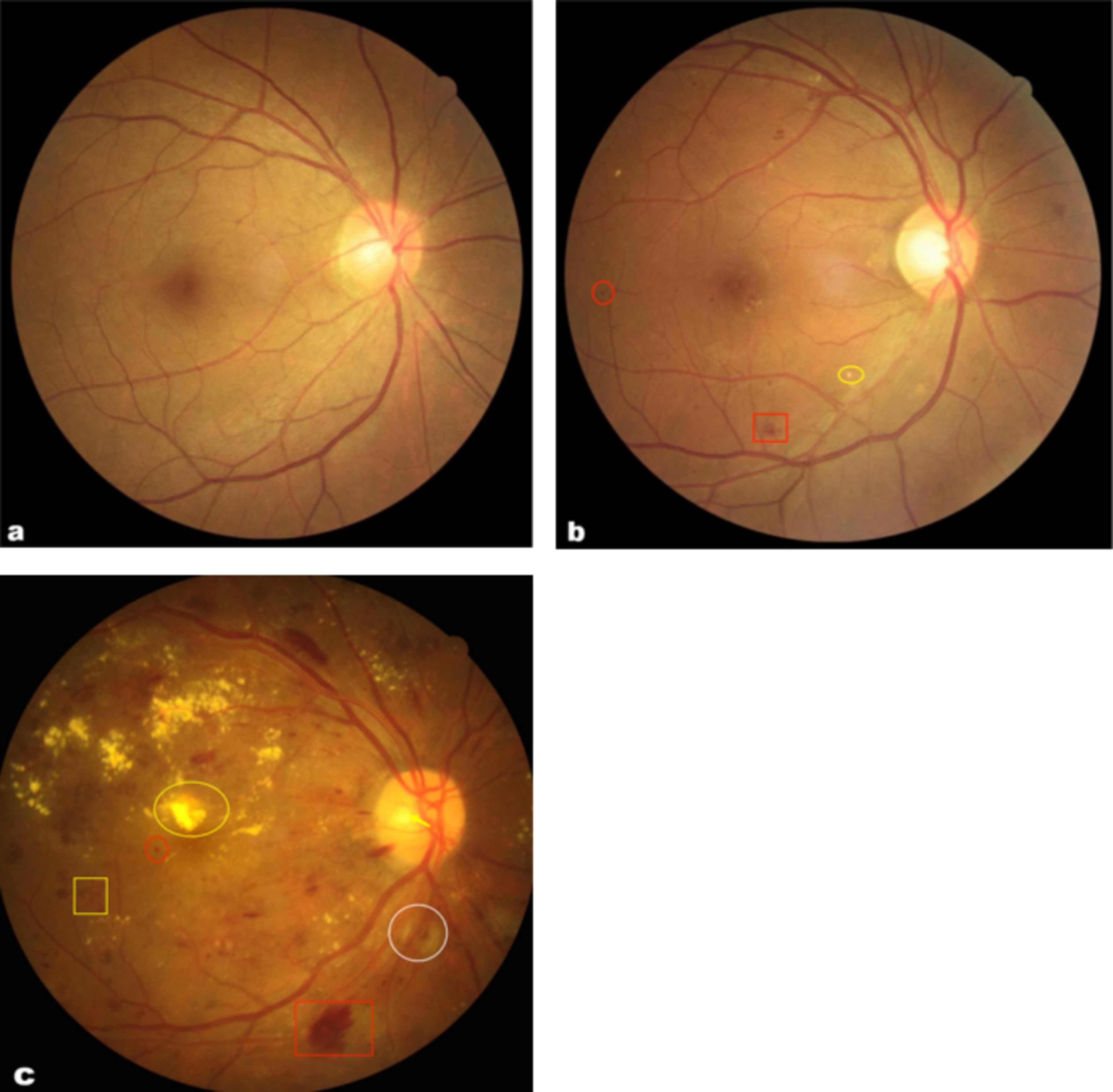

Supplement: Supplementary file 3 — Supplementary Material 3 [file 12886_2023_2925_MOESM3_ESM.jpg]
