# Supplementary material for: Dynamic nomogram prediction model for diabetic retinopathy in patients with type 2 diabetes mellitus
Source: BMC Ophthalmol. 2023 Apr 28;23:186. doi: 10.1186/s12886-023-02925-1 (PMC10142167; doi:10.1186/s12886-023-02925-1)
Supplement: Supplementary file 2 — Supplementary Material 2 [file 12886_2023_2925_MOESM2_ESM.docx]

**[Supplementary](javascript:;) Table 1 Candidate Variables for predicting DR**

| Variables group | Variables type | Variables |
| --- | --- | --- |
| Clinical features | Continuous | Age, Waistline |
|  | Categorical | Grade, Sex, SBP, DBP, BMI |
|  |  | Family history, Diabetes duration, AOO, |
|  |  | Education, Smoking, Drinking, Treatment |
| Medical history | Categorical | Hpertension, Hyperlipimedia, Ketosis, |
|  |  | Kidney disease, CHD, Thyroid disease |
| Laboratory values | Continuous | HBAIC%, FPG, Serum insulin, C-Peptide |
|  |  | HOMA-IR, HOMA-beta, Fructosamine, UA, |
|  |  | BHA, TC, TG, HDL-C, LDL-C, NONHDL, |
|  |  | LP(a), APOA1, APOB, LAP, UREA, CREA, |
|  | Categorical | UACR, MA, Urine sugar |
| CGM parameters | Continuous | SD, MAGE, MG, LAGE, MODD |

AOO: Age of onset of disease; SBP: Systolic blood pressure; DBP: Diastolic blood pressure; FPG: Fasting plasma glucose; HbA1c: Glycosylated hemoglobin; HDL-C: High-density lipoprotein; LDL-C: Low-density lipoprotein; TC: Total cholesterol; TG: Triglycerides; Lp(a): Lipoprotein (a); APOA1: Apolipoprotein A1; APOB: Apolipoprotein B; UREA：CREA: Creatinine; UA: Uric acid; UACR: Urine microalbumin/urine creatinine; MA: Microalbumin; BHA: β-Hydroxybutyric acid; HOMA-IR: Homeostatic model assessment of insulin resistance; HOMA-beta: Homeostatic model assessment of β-cell function; SD: standard deviation; MAGE: Mean amplitude of glucose excursions; LAGE: Largest blood glucose fluctuation; MODD: Mean of daily differences; CHD: coronary heart disease

[**Supplementary**](javascript:;) **Table 2. Correlation of Independent Variables in the training set**

| Variables | B | Std. Error | Beta | *P* value | Tolerance | VIF |
| --- | --- | --- | --- | --- | --- | --- |
| Age | -0.001 | 0.002 | -0.026 | 0.590 | 0.361 | 2.768 |
| Sex | 0.065 | 0.039 | 0.070 | 0.092 | 0.478 | 2.094 |
| Diabetes duration | 0.134 | 0.025 | 0.206 | 0.000 | 0.558 | 1.793 |
| AOO | -0.053 | 0.045 | -0.050 | 0.233 | 0.467 | 2.143 |
| Waistline | 0.004 | 0.003 | 0.076 | 0.151 | 0.299 | 3.340 |
| SBP | 0.012 | 0.022 | 0.018 | 0.602 | 0.684 | 1.462 |
| DBP | 0.024 | 0.021 | 0.039 | 0.260 | 0.688 | 1.453 |
| Education | -0.026 | 0.028 | -0.028 | 0.357 | 0.889 | 1.124 |
| BMI | -0.038 | 0.026 | -0.062 | 0.139 | 0.475 | 2.106 |
| Family history | 0.015 | 0.028 | 0.017 | 0.582 | 0.921 | 1.086 |
| Smoking | -0.011 | 0.036 | -0.012 | 0.768 | 0.544 | 1.837 |
| Drinking | 0.017 | 0.036 | 0.017 | 0.641 | 0.632 | 1.582 |
| Treatment | 0.029 | 0.013 | 0.071 | 0.029 | 0.783 | 1.277 |
| Hypertension | -0.007 | 0.030 | -0.008 | 0.813 | 0.755 | 1.324 |
| Hyperlipimedia | 0.009 | 0.032 | 0.009 | 0.778 | 0.752 | 1.331 |
| Ketosis | -0.025 | 0.037 | -0.023 | 0.493 | 0.764 | 1.309 |
| Kidney disease | 0.013 | 0.036 | 0.012 | 0.709 | 0.75 | 1.333 |
| CHD | -0.018 | 0.040 | -0.014 | 0.651 | 0.825 | 1.212 |
| Thyroid disease | -0.036 | 0.028 | -0.039 | 0.206 | 0.869 | 1.150 |
| HBAIC% | 0.008 | 0.009 | 0.043 | 0.383 | 0.342 | 2.920 |
| FPG | 0.005 | 0.006 | 0.048 | 0.431 | 0.228 | 4.389 |
| Serum insulin | 0.009 | 0.005 | 0.185 | 0.058 | 0.088 | 11.382 |
| C-Peptide | -0.027 | 0.015 | -0.078 | 0.063 | 0.475 | 2.104 |
| HOMA-IR | -0.014 | 0.009 | -0.164 | 0.123 | 0.074 | 13.52 |
| HOMA-beta | 0.000 | 0.000 | -0.041 | 0.167 | 0.946 | 1.057 |
| BHA | 0.000 | 0.000 | -0.040 | 0.415 | 0.343 | 2.916 |
| TC | 0.037 | 0.026 | 0.112 | 0.149 | 0.139 | 7.218 |
| TG | 0.007 | 0.02 | 0.053 | 0.711 | 0.04 | 24.894 |
| HDL-C | -0.014 | 0.021 | -0.021 | 0.507 | 0.866 | 1.155 |
| LDL-C | -0.035 | 0.034 | -0.074 | 0.295 | 0.168 | 5.951 |
| NONHDL | 0.021 | 0.030 | 0.059 | 0.484 | 0.117 | 8.533 |
| LP(a) | 0.000 | 0.001 | 0.006 | 0.839 | 0.895 | 1.118 |
| APOA1 | -0.020 | 0.036 | -0.018 | 0.574 | 0.836 | 1.197 |
| APOB | 0.006 | 0.031 | 0.006 | 0.852 | 0.775 | 1.291 |
| LAP | 0.000 | 0.001 | -0.112 | 0.446 | 0.039 | 25.802 |
| UREA | -0.002 | 0.009 | -0.01 | 0.789 | 0.583 | 1.716 |
| CREA | 0.001 | 0.001 | 0.064 | 0.127 | 0.47 | 2.129 |
| UA | 0.000 | 0.000 | -0.025 | 0.475 | 0.691 | 1.447 |
| UACR | 0.133 | 0.033 | 0.173 | 0.000 | 0.447 | 2.238 |
| MA | -0.027 | 0.062 | -0.018 | 0.664 | 0.477 | 2.095 |
| Urine sugar | 0.086 | 0.032 | 0.091 | 0.008 | 0.714 | 1.401 |
| SD | -0.009 | 0.009 | -0.237 | 0.341 | 0.013 | 74.415 |
| MAGE | 0.001 | 0.001 | 0.045 | 0.525 | 0.169 | 5.914 |
| MG | 0.003 | 0.004 | 0.043 | 0.516 | 0.194 | 5.165 |
| LAGE | 0.001 | 0.002 | 0.116 | 0.568 | 0.020 | 48.987 |
| MODD | 0.001 | 0.001 | 0.032 | 0.382 | 0.633 | 1.579 |

Categorical variables are expressed as frequencies (%), and quantitative variables are expressed as medians (IQR). (AOO: Age of onset of disease; SBP: Systolic blood pressure; DBP: Diastolic blood pressure; FPG: Fasting plasma glucose; HbA1c: Glycosylated hemoglobin; HDL-C: High-density lipoprotein; LDL-C: Low-density lipoprotein; TC: Total cholesterol; TG: Triglycerides; Lp(a): Lipoprotein (a); APOA1: Apolipoprotein A1; APOB: Apolipoprotein B; CREA: Creatinine; UA: Uric acid; UACR: Urine microalbumin/urine creatinine; MA: Microalbumin; BHA: β-Hydroxybutyric acid; HOMA-IR: Homeostatic model assessment of insulin resistance; HOMA-beta: Homeostatic model assessment of β-cell function; SD: standard deviation; MAGE: Mean amplitude of glucose excursions; LAGE: Largest blood glucose fluctuation; MODD: Mean of daily differences; CHD: coronary heart disease)

[**Supplementary**](javascript:;) **Table 3. Univariable logistic regression analysis of patients with concurrent DR in the training set**

| Variables | *β* | OR (95%CI) | P |
| --- | --- | --- | --- |
| Diabetes duration (years, vs. ≤10) |  |  |  |
| 10-19 | 0.90 | 2.45 (1.79-3.35) | <0.001 |
| 20-29 | 1.72 | 5.61 (3.55-8.95) | <0.001 |
| ≥30 | 1.57 | 4.79 (1.42-16.84) | 0.011 |
| AOO (years)  >40 vs. <40 | -0.53 | 0.59 (0.43-0.79) | <0.001 |
| Treatment (vs. no treatment) |  |  | <0.001 |
| Insulin | 1.56 | 4.74 (3.03-7.57) | <0.001 |
| Oral antidiabetic drugs | 0.89 | 2.43 (1.59-3.78) | <0.001 |
| Combination therapy | 1.39 | 4.03 (2.59-6.37) | <0.001 |
| SBP (mmHg)  ≥140 vs. <140 | 0.60 | 1.82 (1.25-2.66) | 0.002 |
| Hypertension | 0.34 | 1.41 (1.07-1.85) | 0.013 |
| Kidney disease | 0.66 | 1.94 (1.43-2.63) | <0.001 |
| Education  High school or above | -0.29 | 0.75 (0.57-0.98) | 0.035 |
| C-peptide (ng/ml) | -0.18 | 0.84 (0.75-0.94) | 0.002 |
| TC (mmol/L) | 0.12 | 1.13 (1.02-1.25) | 0.014 |
| NONHDL (mmol/L) | 0.13 | 1.14 (1.02-1.26) | 0.018 |
| Lpa (mg/dL) | 0.01 | 1.01 (1.01-1.02) | 0.042 |
| UREA (mmol/L) | 0.096 | 1.10 (1.02-1.28) | 0.009 |
| CREA (µmol/L) | 0.001 | 1.01 (1.00-1.01) | 0.006 |
| UACR (mg/g cr) |  |  |  |
| 30-300 | 0.89 | 2.25 (1.73-3.44) | <0.001 |
| >300 | 1.69 | 5.42 (3.27-9.13) | <0.001 |
| MA (mg/dl) |  |  |  |
| 20-200 | 1.35 | 3.87 (2.36-6.39) | <0.001 |
| >200 | 1.58 | 4.83 (1.18-23.70) | 0.032 |
| Urine sugar  positive | 0.39 | 1.48 (1.11-1.99) | 0.009 |

Categorical variables are expressed as frequencies (%), and quantitative variables are expressed as medians (IQR). (AOO: Age of onset of disease; SBP: Systolic blood pressure; TC: total cholesterol; Lp(a): Lipoprotein (a);CREA: Creatinine; UA: Uric acid; UACR: Urine microalbumin/urine creatinine; MA: Microalbumin)
