# Supplementary material for: Dynamic nomogram prediction model for diabetic retinopathy in patients with type 2 diabetes mellitus
Source: BMC Ophthalmol. 2023 Apr 28;23:186. doi: 10.1186/s12886-023-02925-1 (PMC10142167; doi:10.1186/s12886-023-02925-1)
Supplement: Supplementary file 4 — Supplementary Material 4 [file 12886_2023_2925_MOESM4_ESM.docx]

**Supplementary materials**

**Supplementary Figure 1.** a-b. Determination of the number of factors by the LASSO analysis

**Supplementary Figure 2.** Color fundus photography of the posterior pole of the right eye. a. Fundus photo of T2DM; b. Fundus photo of NPDR: Posterior pole multiple intraretinal hemorrhages (red rectangle), microangiomas (red circle), rigid exudate (yellow circle); c. Fundus photo of PDR: There are many rigid exudates (yellow circle) in the posterior pole, intraretinal hemorrhage (red rectangle), microangioma (red circle), cotton wool spots (white circle), intraretinal microvascular abnormalities (yellow rectangle), and optic disc neovascularization (yellow arrow).
